# Effects and Implementation of a Mindfulness and Relaxation App for Patients With Cancer: Mixed Methods Feasibility Study

**DOI:** 10.2196/16785

**Published:** 2021-01-13

**Authors:** Michael Mikolasek, Claudia Margitta Witt, Jürgen Barth

**Affiliations:** 1 Institute for Complementary and Integrative Medicine University Hospital Zurich and University of Zurich Zurich Switzerland; 2 Institute for Social Medicine, Epidemiology and Health Economics, Charité Universitätsmedizin Berlin Berlin Germany; 3 Center for Integrative Medicine University of Maryland School of Medicine Baltimore, MD United States

**Keywords:** mobile app, mobile phone, mindfulness, relaxation, cancer, qualitative research, implementation science, mHealth, evaluation study, patient compliance, patient participation, patient preference

## Abstract

**Background:**

Cancer diagnosis and cancer treatment can cause high levels of distress, which is often not sufficiently addressed in standard medical care. Therefore, a variety of supportive nonpharmacological treatments have been suggested to reduce distress in patients with cancer. However, not all patients use these interventions because of limited access or lack of awareness. To overcome these barriers, mobile health may be a promising way to deliver the respective supportive treatments.

**Objective:**

The aim of this study is to evaluate the effects and implementation of a mindfulness and relaxation app intervention for patients with cancer as well as patients’ adherence to such an intervention.

**Methods:**

In this observational feasibility study with a mixed methods approach, patients with cancer were recruited through the web and through hospitals in Switzerland. All enrolled patients received access to a mindfulness and relaxation app. Patients completed self-reported outcomes (general health, health-related quality of life, anxiety, depression, distress, mindfulness, and fear of progression) at baseline and at weeks 4, 10, and 20. The frequency of app exercise usage was gathered directly through the app to assess the adherence of patients. In addition, we conducted interviews with 5 health professionals for their thoughts on the implementation of the app intervention in standard medical care. We analyzed patients’ self-reported outcomes using linear mixed models (LMMs) and qualitative data with content analysis.

**Results:**

A total of 100 patients with cancer (74 female) with a mean age of 53.2 years (SD 11.6) participated in the study, of which 25 patients used the app regularly until week 20. LMM analyses revealed improvements in anxiety (*P*=.04), distress (*P*<.001), fatigue (*P*=.01), sleep disturbance (*P*=.02), quality of life (*P*=.03), and mindfulness (*P*<.001) over the course of 20 weeks. Further LMM analyses revealed a larger improvement in distress (*P*<.001), a moderate improvement in anxiety (*P*=.001), and a larger improvement in depression (*P*=.03) in patients with high levels of symptoms at baseline in the respective domains. The interviews revealed that the health professionals perceived the app as a helpful addition to standard care. They also made suggestions for improvements, which could facilitate the implementation of and adherence to such an app.

**Conclusions:**

This study indicates that a mindfulness and relaxation app for patients with cancer can be a feasible and effective way to deliver a self-care intervention, especially for highly distressed patients. Future studies should investigate if the appeal of the app can be increased with more content, and the effectiveness of such an intervention needs to be tested in a randomized controlled trial.

## Introduction

### Background

Cancer diagnosis and subsequent medical treatments can cause high levels of distress [1-4]. However, adequate psychological support for patients with cancer is often lacking in standard medical care [5,6]. Therefore, a variety of supportive treatments have been suggested to reduce distress in patients with cancer, such as mind-body medicine (MBM) [7]. MBM combines various effective treatments such as mindfulness meditation, relaxation, yoga, and tai chi [7,8]. Such MBM treatments can have beneficial effects on cancer-related symptoms, such as pain, fatigue, and sleep disturbance [9-11]. Furthermore, MBM treatments can have beneficial effects on the quality of life of patients with cancer [12-14]. These treatments can be provided through guided MBM programs for patients with cancer, where the patients learn various exercises (eg, physical exercises, relaxation, and stress reduction) and are encouraged to practice these newly learned exercises at home [15,16].

However, the uptake of supportive treatments in distressed patients with cancer is moderate [17]. Barriers for the uptake of such treatments include stigmatization, unawareness of such interventions, or limited access [18,19]. This is problematic because untreated, elevated levels of distress can lead to additional negative effects, such as reduced quality of life, daily functioning, and lower adherence to medical treatment [20,21]. Access can be restricted, for instance, because of geographical distance, lack of treatment providers or knowledge thereof, and financial constraints [22-24]. To overcome these limitations in access, eHealth and mobile health (mHealth) interventions have been proposed. eHealth is defined more broadly as the delivery of health services or information through the internet and related technologies [25], whereas mHealth uses mobile technologies such as smartphones for the delivery of health services [26]. So far, research indicates that eHealth interventions with mindfulness or relaxation components can have beneficial effects on health outcomes in various patient populations [27-29]. However, eHealth studies focusing on patients with cancer have shown inconsistent results [30,31]. Nonetheless, eHealth interventions seem promising because they can have positive effects on the well-being of patients with cancer [31].

Although mHealth interventions have some advantages over web-based eHealth interventions (eg, more flexible access because of mobility, the possibility of reaching a large number of patients because of the large popularity of smartphones), little is known about the best practices for the implementation of mHealth interventions [32,33]. In addition, mHealth research so far indicates that the adoption of mHealth interventions by health professionals and patients can be inhibited by various factors, such as perceived usefulness and ease of use [34,35]. Furthermore, there is a lack of mHealth studies with mindfulness or relaxation-based interventions [27]. Therefore, we developed a research app to conduct a feasibility study of a mindfulness- and relaxation-based mHealth intervention for patients with cancer [36]. The app included 3 exercises, namely, mindfulness meditation, guided imagery, and progressive muscle relaxation.

### Objectives

The aim of this study is to assess the feasibility of this mHealth intervention using the Reach, Effectiveness, Adoption, Implementation, and Maintenance (RE-AIM) evaluation framework, which was developed for the evaluation of public health interventions [37]. Although the results for the reach of the dimensions, adoption over the course of 10 weeks, and maintenance were published elsewhere [36], the present analyses focus on the 3 dimensions of effectiveness, adoption, and implementation over the course of 20 weeks to assess the pre-post effects of the app on a variety of health outcomes and adherence to the app intervention. In doing so, we investigate whether such an app may be a beneficial, supportive care tool for patients with cancer.

## Methods

### Study Design

For this feasibility study, we used a mixed methods approach. For quantitative data, we assessed 4 paper-and-pencil questionnaires that were sent to patients with cancer at baseline and at weeks 4, 10, and 20. Demographics and patient characteristics were assessed at baseline, and health outcomes (physical, mental, and social health, health-related quality of life, anxiety, depression, distress, mindfulness, and fear of progression) were assessed over the 4 time points. Qualitative data consisted of semistructured interviews with 5 health professionals. In those interviews, we inquired about health professionals’ perspectives on a mindfulness- and relaxation-based mHealth intervention for patients with cancer and its implementation in standard medical care. To receive feedback from different health professionals, we conducted 2 face-to-face group interviews (1 interview with 2 nursing experts and the second interview with 2 psychologists providing MBM treatment for patients with cancer) and 1 individual interview with an oncologist. All interviewees received access to the app before the interview and could test the app. The interviewer also demonstrated the app and its content to the interviewees before the interview started.

To assess the feasibility of our mHealth intervention, we used the RE-AIM implementation science framework [37]. Ethical approval for the study was granted in April 2016 by the cantonal ethics committee Zurich (BASEC-Nr. 2016-00258), and we registered the study in the German Clinical Trials Register (DRKS00010481).

### Participants

Patients were eligible if they (1) had any cancer diagnosis at any stage of cancer, (2) were aged 18 years or older, and (3) owned either an iPhone (Apple Inc). or an Android-based smartphone with at least a weekly connection to the internet. Patients were excluded if they had suicidal ideation or insufficient German language skills, if they intended to move to another country, or if they had insufficient knowledge on how to use a smartphone. The patient recruitment process is described in detail elsewhere [36]. For the interviews with health professionals, we invited experts (an oncologist, nursing experts, and psychologists) from the University Hospital Zurich, who provide health care for patients with cancer.

### App Intervention

All enrolled patients received the mindfulness and relaxation app, which was specifically developed for this study and only available for patients participating in the study. The app could be downloaded in the Apple iTunes store and Google Play Store for Android devices and accessed with a code, which was provided to the patients after study inclusion. The app offered 3 exercises: mindfulness meditation, guided imagery, and progressive muscle relaxation. The exercises were included in the app as audio files with a duration of approximately 15 minutes each, and the patients could choose between a female or male narrator. Patients were free to choose which exercises they wanted to use and how often they wanted to practice. However, we recommended to the patients to use an exercise of their choice on a daily basis, ideally 5 times per week. To help patients practice regularly, the app included an optional notification feature that patients could set up to receive a daily push notification on the mobile device, reminding them to practice at an individually set time. Information about the use of exercises (exercise type, date, and start and end times) was saved in the backend and was only accessible to the researchers as an XML log file. More information about the app is presented in a previously published paper [36].

### Outcomes

#### Effects

As we conducted a single-arm study without a control group, we were not able to assess the effectiveness of the app intervention. Therefore, for the RE-AIM dimension effectiveness, we looked into pre-post effects in a variety of health outcomes relevant to patients with cancer. We assessed physical, mental, and social health using the Patient-Reported Outcomes Measurement Information System (PROMIS 29) [38]. PROMIS 29 is a 29-item scale assessing 7 health domains: physical function (Cronbach α=.81), fatigue (Cronbach α=.94), pain interference (Cronbach α=.96), depressive symptoms (Cronbach α=.85), anxiety (Cronbach α=.81), ability to participate in social roles and activities (Cronbach α=.88), and sleep disturbance (Cronbach α=.86) with 4 items, each on a 5-point scale, and pain intensity with a single item on a 10-point numeric rating scale.

For the assessment of health-related quality of life for patients with cancer, we administered the Functional Assessment of Cancer Therapy—General (FACT-G) [39,40]. The FACT-G consists of 4 subscales: physical well-being (Cronbach α=.85), social well-being (Cronbach α=.76), emotional well-being (Cronbach α=.70), and functional well-being (Cronbach α=.79), measured with 27 items on a 5-point scale. A higher score indicates a better quality of life.

For the assessment of distress, we administered the Distress Thermometer [41]. The Distress Thermometer is a numeric rating scale, ranging from 0 to 10. A score of 5 or higher is considered to indicate clinically relevant distress [42].

For the assessment of mindfulness, we administered the short version of the Freiburg Mindfulness Inventory (FMI) [43]. The FMI (Cronbach α=.87) assesses mindfulness with 14 items on a 4-point scale, with a higher score indicating higher mindfulness.

We measured anxiety and depression using the Hospital Anxiety and Depression Scale (HADS). The HADS assesses 7 items for the subscales anxiety (Cronbach α=.79) and depression (Cronbach α=.67) on a 4-point scale, with a maximum score of 21 for each subscale. A score of up to 7 is considered normal, a score between 8 and 11 is considered borderline, and a score above 11 is considered caseness [44].

For the assessment of fear of progression, we administered the Fear of Progression Questionnaire-Short Form (FoP-Q-SF) [45]. The FoP-Q-SF (Cronbach α=.81) consists of 12 items with a 5-point scale. A higher score indicates a greater fear of progression.

We assessed PROMIS 29, FACT-G, and FMI at baseline and at weeks 4, 10, and 20 and HADS, FoP-Q-SF, and Distress Thermometer at baseline and at weeks 10 and 20. We defined a continuous app user as a patient who regularly used the app exercises (at least one exercise per week). We counted an exercise as completed if the patient played the exercise audio file for at least 10 minutes of the total time of 15 minutes. We defined an intervention dropout as a patient who stopped using the exercises for 4 consecutive weeks because regular practice might be a prerequisite for a beneficial intervention. We defined the first week when the patient stopped using the exercises as a dropout week. A patient who never used an app exercise was counted as a week 1 intervention dropout.

#### Adoption

For the RE-AIM dimension adoption, we looked at the number of completed app exercises over 20 weeks and app exercise preferences. We reported the median of completed app exercises by all enrolled patients per week as well as the median of completed app exercises by continuous app users. For exercise preferences, we reported frequencies of used exercises for all enrolled patients, stratified by gender of the patient and the narrator.

#### Implementation

For the RE-AIM dimension implementation, we reported results from interviews with health professionals regarding their opinion on the implementation of the app intervention in addition to standard medical care. In the interviews, we inquired about the general impression regarding the app, implementation of the app as an addition to standard medical care, and suggestions for improvements.

### Sample Size

One aspect evaluated in our feasibility study was the characteristics and number of patients with cancer who participated in the study (evaluation dimension reach), which was reported previously [36]. Therefore, we did not perform an a priori analysis to determine the required sample size for adequate power. However, we aimed to recruit at least 100 patients, which is sufficient to achieve 80% power for a two-tailed *t* test with an α level set at .05 and a small effect size of Cohen *d* of 0.28.

### Data Analysis

#### Quantitative Data

All printed case report forms were entered by trained researchers into the electronic database REDCap (Research Electronic Data Capture), which was hosted at the University Hospital Zurich. All analyses were carried out in SPSS version 25.0 (IBM Corp).

For baseline characteristics of patients, we used descriptive statistics (frequencies and percentages for categorical variables and mean and SD for continuous variables). For the analyses of pre-post effects, we used linear mixed models (LMMs) to analyze changes over time (baseline, week 4, week 10, and week 20) in health outcomes as well as differences between continuous app users and intervention dropouts in health outcomes. All patients who provided baseline data were included in the analyses, and because we used LMMs, patients with missing data in weeks, 4, 10, and 20 questionnaires were included. The dependent variables were the 7 PROMIS 29 domains, FACT-G, HADS subscales anxiety and depression, Distress Thermometer, FMI, and FoP-Q-SF. Furthermore, we looked at the changes in the respective health outcomes for subsamples with high distress (Distress Thermometer score ≥5), high anxiety (HADS anxiety score of ≥8), and high depression (HADS depression score of ≥8). As a covariance type, we used an autoregressive covariance structure (AR1). Time was included as a fixed effect. For group analyses, (continuous app users vs intervention dropouts), we added group and time-by-group as fixed effects. Hedge *g* effect sizes were calculated as mean differences (baseline and week 20) divided by pooled SDs for each health outcome of interest.

#### Qualitative Data

For the dimension implementation, we recorded the interviews and transcribed the interviews verbatim. We used thematic coding for structuring the interviews using MAXQDA 11 (VERBI Software), and we used content analysis according to Mayring [46].

## Results

### Patient Characteristics

**Figure 1:**
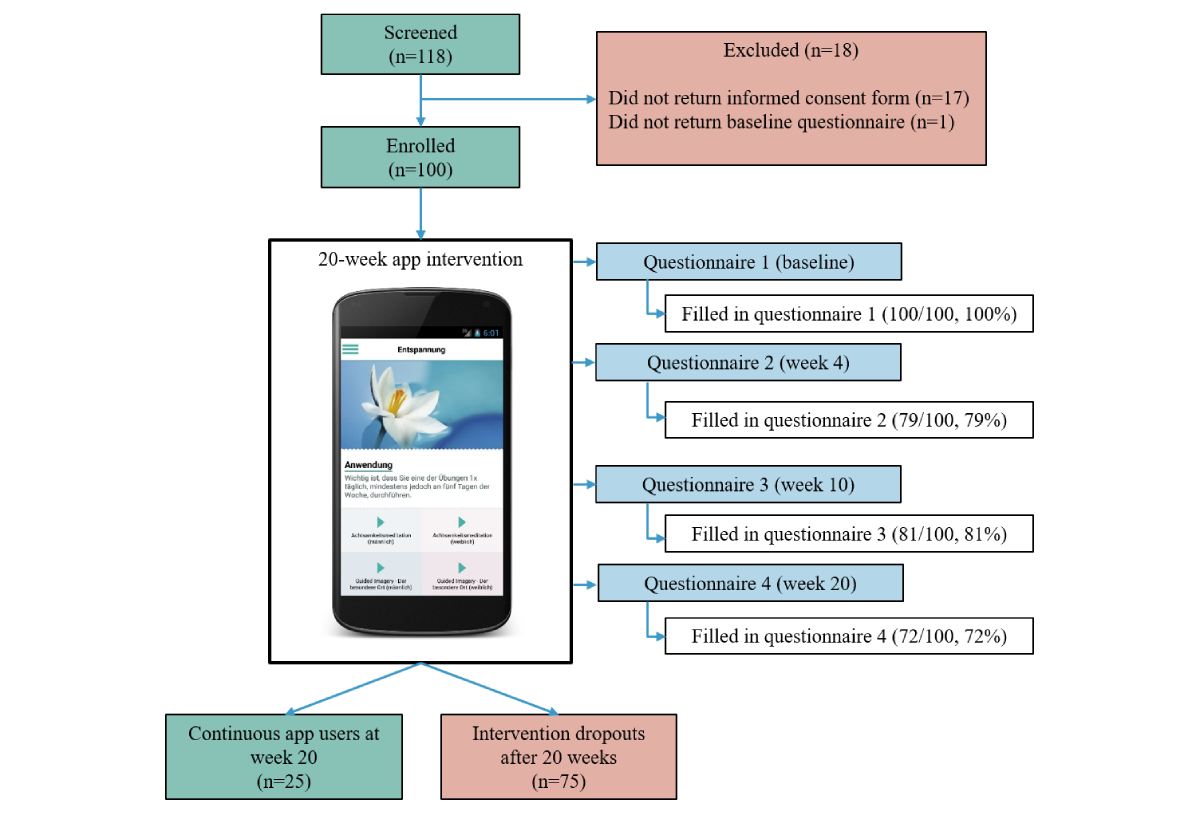
Flowchart.

Between June 2016 and December 2018, we were able to recruit 100 patients with cancer, all of whom provided baseline information. At week 20, 72 (72%) patients completed questionnaire 4 (Figure 1). Baseline characteristics of all enrolled patients (N=100) as well as subsamples of patients with high distress (62/100, 62%), high anxiety (35/100, 26%), and high depression (20/100, 20%) are summarized in Table 1. Most patients (74/100, 74%) were female. The mean age of all patients was 53.24 (SD 11.55) years, ranging from 23 to 84 years. Patients predominantly owned an iPhone smartphone (67/100, 67%), whereas 30 patients (30/100, 30%) owned an Android smartphone, and a few (3/100, 3%) owned both.

**Table 1:** Demographics for the total sample and high distress, high depression, and high anxiety subsamples.

Patient demographics		Total sample (N=100)	High distress^a^ subsample (n=62)	High anxiety^b^ subsample (n=35)	High depression^c^ subsample (n=20)
**Gender, n (%)**					
	Female	74 (74)	48 (77)	26 (74)	15 (75)
	Male	26 (26)	14 (23)	9 (26)	5 (25)
Age (years), mean (SD)		53.24 (11.55)	52.74 (10.67)	51.22 (10.67)	51.74 (11.63)
**Type of cancer, n (%)**					
	Breast cancer	39 (39)	27 (44)	18 (51)	8 (40)
	Colon cancer	9 (9)	7 (11)	2 (6)	3 (15)
	Ovarian or cervical cancer	6 (6)	3 (5)	2 (6)	0 (0)
	Lung cancer	6 (6)	3 (5)	0 (0)	1 (5)
	Others	40 (40)	22 (35)	13 (37)	8 (40)
**Status of cancer treatment, n (%)**					
	Total removal	46 (46)	33 (53)	24 (69)	11 (55)
	Recurrence or incomplete removal	25 (25)	15 (24)	6 (17)	5 (25)
	Uncertain	3 (3)	1 (2)	1 (3)	2 (10)
	Other	26 (26)	13 (21)	4 (11)	4 (20)
**Highest education, n (%)**					
	Primary school	3 (3)	2 (3)	2 (6)	0 (0)
	Apprenticeship	22 (22)	16 (26)	5 (14)	5 (25)
	Secondary education	41 (41)	21 (34)	14 (40)	7 (35)
	University degree	33 (33)	22 (35)	14 (40)	7 (35)
	Unknown	1 (1)	1 (2)	0 (0)	1 (5)

^a^Distress Thermometer score ≥5.

^b^Hospital Anxiety and Depression Scale anxiety score ≥8.

^c^Hospital Anxiety and Depression Scale depression score ≥8.

### Effects

The health outcome values at baseline and at week 20 as well as effect sizes for the total sample and the high distress, high anxiety, and high depression subsamples are presented in Table 2. Baseline distress was 5.29 (SD 2.31); therefore, patients were on average above an assumed clinically relevant threshold of 5, with 62% of patients (62/100) reporting a distress level of 5 or higher. At week 20, distress decreased to an average of 4.1 (SD 2.12; Hedge *g*=0.53). The mean HADS anxiety score at baseline was 6.88 (SD 3.50) and dropped to 6.31 (SD 3.78; Hedge *g*=0.16) at week 20. Overall, 35% (35/100) of patients reported an elevated HADS anxiety score (≥8) at baseline (mean 10.71, SD 1.95), which dropped to 8.85 (SD 3.50; Hedge *g*=0.68) at week 20. For HADS depression, the mean score at baseline was 4.96 (SD 2.78) and dropped to 4.55 (SD 3.31; Hedge *g*=0.14) at week 20. Overall, 20% (20/100) of patients reported an elevated HADS depression score (≥8) at baseline (mean 9.00, SD 1.12), which dropped to 8.85 (SD 3.50; Hedge *g*=0.61) at week 20. For the remaining measures without a proposed threshold (PROMIS, FACT-G, FMI, and FoP-Q-SF), changes from baseline to week 20 were small, with Hedges *g* effect sizes ranging from 0.04 to 0.33.

**Table 2:** Mean values of health outcomes at baseline and week 20, response rate (n), and effect sizes (N=100).

Sample and outcome		Baseline		Week 20		Hedges *g* (95% CI)
		Mean (SD)	n	Mean (SD)	n	
**Total sample**						
	HADS^a^ anxiety	6.88 (3.50)	99	6.31 (3.78)	71	−0.16 (−0.46 to 0.15)
	HADS depression	4.96 (2.78)	100	4.55 (3.31)	71	−0.14 (−0.44 to 0.17)
	Distress	5.29 (2.31)	99	4.10 (2.12)	71	−0.53 (−0.84 to 0.22)
	PROMIS physfunct^b^	46.55 (6.54)	99	46.30 (7.32)	71	−0.04 (−0.34 to 0.27)
	PROMIS anxiety^c^	55.97 (6.46)	99	55.01 (6.83)	70	−0.15 (−0.45 to −0.16)
	PROMIS depression^d^	55.20 (6.81)	100	53.88 (7.81)	71	−0.18 (−0.49 to 0.12)
	PROMIS fatigue^e^	56.11 (9.23)	99	52.40 (10.31)	70	−0.38 (−0.69 to −0.07)
	PROMIS sleep^f^	51.44 (8.85)	100	49.52 (8.02)	70	−0.23 (−0.53 to 0.08)
	PROMIS social^g^	48.42 (7.64)	99	49.84 (7.87)	71	0.18 (−0.12 to 0.49)
	PROMIS pain^h^	52.88 (9.10)	97	51.96 (9.38)	70	0.10 (−0.41 to 0.21)
	FACT-G^i^	75.54 (13.85)	99	79.62 (14.81)	70	0.29 (−0.02 to 0.59)
	FMI^j^	38.46 (6.62)	96	41.80 (6.42)	69	0.51 (−0.20 to 0.83)
	FoP^k^	31.33 (7.83)	93	30.28 (7.99)	64	−0.13 (−0.45 to 0.19)
**High distress^l^**						
	Distress	6.79 (1.36)	62	4.39 (2.19)	46	−1.36 (−1.79 to −0.94)
**High anxiety^m^**						
	HADS anxiety	10.71 (1.95)	35	8.85 (3.50)	26	−0.69 (−1.20 to −0.16)
**High depression^n^**						
	HADS depression	9.00 (1.12)	20	7.47 (3.52)	17	−0.61 (−1.27 to 0.05)

^a^HADS: Hospital Anxiety Depression Scale; negative effect=improvement.

^b^PROMIS physfunct: Patient-Reported Outcomes Measurement Information System Physical Function; positive effect=improvement.

^c^PROMIS anxiety: Patient-Reported Outcomes Measurement Information System Anxiety; negative effect=improvement.

^d^PROMIS depression: Patient-Reported Outcomes Measurement Information System Depression; negative effect=improvement.

^e^PROMIS fatigue: Patient-Reported Outcomes Measurement Information System Fatigue; negative effect=improvement.

^f^PROMIS sleep: Patient-Reported Outcomes Measurement Information System Sleep Disturbance; negative effect=improvement.

^g^PROMIS social: Patient-Reported Outcomes Measurement Information System Ability to Participate in Social Roles and Activities; positive effect=improvement.

^h^PROMIS pain: Patient-Reported Outcomes Measurement Information System Pain Interference; negative effect=improvement.

^i^FACT-G: Functional Assessment of Cancer Therapy—General; positive effect=improvement.

^j^FMI: Freiburg Mindfulness Inventory; positive effect=improvement.

^k^FoP: Fear of Progression; negative effect=improvement.

^l^Distress Thermometer score ≥5; negative effect=improvement; n=62.

^m^HADS anxiety score ≥8; negative effect=improvement; n=35.

^n^HADS depression score ≥8; negative effect=improvement; n=20.

The results for effects over time are presented in Table 3. LMM analyses revealed that there was a significant decrease over time in distress (*P*<.001), fatigue (*P*=.01), sleep disturbance (*P*=.02), and anxiety (*P*=.04) measured with the HADS. Furthermore, there was a significant increase in quality of life (*P*=.03) and mindfulness (*P*<.001). No significant effects were found for physical functioning, anxiety measured with PROMIS, depression, ability to participate in social roles and activities, and fear of progression. LMM analyses for the subsamples revealed that distress decreased significantly in the high distress subsample (*P*<.001), anxiety decreased significantly in the high anxiety subsample (*P*=.001), and depression decreased significantly in the high depression subsample (*P*=.03). Dose-response analyses using LMMs with group-by-time revealed no significant results.

**Table 3:** Linear mixed models: estimates of fixed effect of time on health outcomes from baseline to week 20.

Sample and dependent variable		Estimates of fixed effects (time)		
		Estimate (95% CI)	*t* test (*df*)	*P* value
**Total sample (N=100)**				
	HADS^a^ anxiety	−0.40 (−0.79 to −0.01)	−2.04 (201.95)	.04
	HADS depression	−0.29 (−0.62 to 0.04)	−1.71 (206.42)	.09
	Distress	−0.41 (−0.62 to −0.21)	−3.96 (325.86)	<.001
	PROMIS physfunct^b^	−0.13 (−0.68 to 0.43)	−.45 (318.35)	.66
	PROMIS anxiety^c^	−0.46 (−1.09 to 0.18)	−1.42 (325.74)	.16
	PROMIS depression^d^	−0.52 (−1.11 to 0.07)	−1.72 (324.81)	.09
	PROMIS fatigue^e^	−1.15 (−2.02 to −0.28)	−2.61 (324.73)	.01
	PROMIS sleep^f^	−0.85 (−1.55 to −0.15)	−2.39 (322.65)	.02
	PROMIS social^g^	0.43 (−0.15 to 1.01)	1.45 (314.63)	.15
	PROMIS pain^h^	−0.14 (−0.94 to 0.66)	−.34 (322.51)	.74
	FACT-G^i^	1.13 (0.10 to 2.15)	2.16 (307.58)	.03
	FMI^j^	1.11 (0.62 to 1.59)	4.46 (300.46)	<.001
	FoP^k^	−0.68 (−1.56 to .20)	−1.52 (180.05)	.13
**High distress^l^ (n=62)**				
	Distress	−0.81 (−1.05 to −0.57)	−6.64 (200.45)	<.001
**High anxiety^m^ (n=35)**				
	HADS anxiety	−1.13 (−1.77 to −0.48)	−3.47 (81.69)	.001
**High depression^n^ (n=20)**				
	HADS depression	−0.87 (−1.65 to −0.09)	−2.23 (47.99)	.03

^a^HADS: Hospital Anxiety Depression Scale.

^b^PROMIS physfunct: Patient-Reported Outcomes Measurement Information System Physical Function.

^c^PROMIS anxiety: Patient-Reported Outcomes Measurement Information System Anxiety.

^d^PROMIS depression: Patient-Reported Outcomes Measurement Information System Depression.

^e^PROMIS fatigue: Patient-Reported Outcomes Measurement Information System Fatigue.

^f^PROMIS sleep: Patient-Reported Outcomes Measurement Information System Sleep Disturbance.

^g^PROMIS social: Patient-Reported Outcomes Measurement Information System Ability to Participate in Social Roles and Activities.

^h^PROMIS pain: Patient-Reported Outcomes Measurement Information System Pain Interference.

^i^FACT-G: Functional Assessment of Cancer Therapy—General.

^j^FMI: Freiburg Mindfulness Inventory.

^k^FoP: Fear of Progression.

^l^Distress Thermometer score ≥5.

^m^HADS anxiety score ≥8.

^n^HADS depression score ≥8.

### Adoption

**Figure 2:**
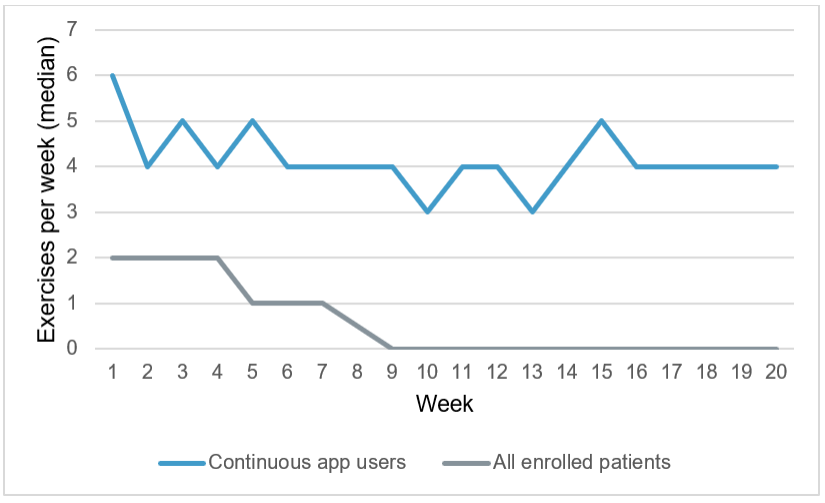
Completed app exercises by all enrolled patients (N=100) and by continuous app users (n=25) per week (median).

According to our definition, 25% (25/100) of all enrolled patients used the app continuously (ie, at least one completed exercise per week) at week 20 of the intervention. The average number (median) of completed exercises during the 20-week intervention for all patients as well as continuous app users is presented in Figure 2. Across all patients, the median of completed exercises was 2 during the first week and dropped to 0 at week 9. For continuous app users, who completed an app exercise at least once per week until week 20, the median of completed exercises at week 1 was 6. For the subsequent weeks up to week 20, the median of completed exercises varied between a median of 3 and 5 for the continuous app users.

**Figure 3:**
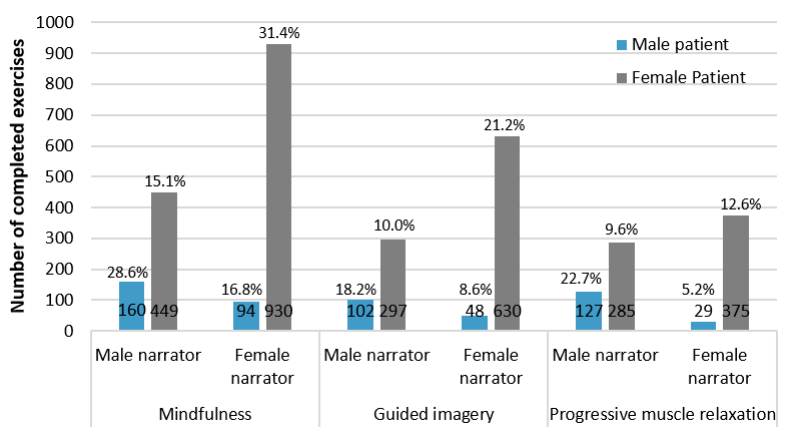
Completed exercises (3526) of all patients (N=100) over 20 weeks by type (mindfulness meditation, guided imagery, and progressive muscle relaxation), gender of patient (male and female), and sex of narrator (male and female). Percentages refer to the total number of exercises per gender.

The percentage of completed exercises is presented in Figure 3. All patients together completed 3526 exercises. Mindfulness meditation was used most often, with a total of 1633 completed exercises (46.31%), followed by guided imagery with 1077 completed exercises (30.55%). Progressive muscle relaxation was used least frequently, with 816 completed exercises (23.14%). In both mindfulness meditation and guided imagery, the female narrator voice was preferred.

Furthermore, female patients showed a preference for exercises with a female narrator (1935 completed exercises with a female narrator vs 1031 completed exercises with a male narrator). However, male patients preferred exercises with a male narrator (389 completed exercises with a male narrator vs 171 completed exercises with a female narrator). The probability of choosing the same sex in audio files is therefore increased for women by 87% and for men by 127%, which corresponds to a 2-fold higher preference for the same sex as the narrator.

### Implementation

A total of 5 health professionals took part in an interview: 2 female nursing experts (one from an inpatient unit and the other from an outpatient oncology unit), 2 female MBM psychologists, and 1 male oncologist. Interviews were conducted between January and March 2018 and lasted for an average of 45 minutes (SD 9.54). The qualitative analysis of the interviews yielded 4 themes: (1) general impression of the app, (2) suggestions for improvement, (3) implementation in standard care, and (4) experience with recommending the app to patients.

Overall, the general impression of the app was positive. For instance, the oncologist summarized his impression of the app as follows:

I think [the app] is a very helpful thing because it is relatively easy [to use]. You can test it. You can try it and if you like it, you can integrate it relatively easy into everyday life. I think it is very practical. It is a practical thing and if patients are interested, I also see that they take it up willingly.

All health professionals perceived the app as appealing, clearly structured, and as a helpful supportive tool. In addition, the MBM psychologists liked the app as an addition to the 10-week face-to-face MBM course and appreciated the app as a good self-help tool complementing the course. The oncologist also stated that many patients with cancer look for something they can use to add to standard care and an app can provide a low threshold aid. As a negative aspect, a nursing expert stated that a smartphone is required and not every patient possesses such a device.

All health experts made various suggestions for improving the app. A shared opinion was that the content of the app (ie, number and variety of exercises) could be increased, as over an extended period, patients might get bored with a choice limited to 3 exercises. A nursing expert suggested that a new exercise could, for instance, be unlocked after completing the same exercise several times. An MBM psychologist suggested that every week, a different selection of exercises could be activated with alternating topics such as meditation, relaxation, self-compassion, or body exercises. In addition, the inclusion of exercises with different degrees of complexity was suggested. An MBM psychologist stated that exercises for beginners (eg, more detailed instructions, fewer moments of silence) as well as exercises for patients experienced in mindfulness and relaxation could be added. MBM psychologists and nursing experts also recommended that some exercises should be accompanied by soothing background music because longer periods of silence might be uncomfortable for some patients. They also recommended exercises with various lengths of time so that patients had more flexibility if they were facing time constraints or if they were too impatient for longer exercises. The oncologist mentioned that adding exercises specifically for sleep disorders might be a good addition to the app, especially for inpatients, because poor sleep in hospitals is very common. As an additional topic that could be added, he mentioned body exercises such as yoga. An MBM psychologist mentioned that an app mirroring the MBM course more closely would be great:

If I could make a wish, then I would say, it would be totally cool to have an accompanying Mind Body Medicine app. That is to say that a lot of exercises—not all of them—but a lot of exercises we do [could be added to the app]. Possibly also guided body exercises. That would be totally cool.

The interviewees mentioned several factors that could influence the implementation of a mindfulness- and relaxation-based app into standard care. Both nursing experts and one of the MBM psychologists stated that the time point when the information of the app is delivered to the patient might be important. These health professionals mentioned that the patients were bombarded with information during the first consultation or during the first day when a patient enters the hospital and additional information about the app might overwhelm some patients. The outpatient nursing expert also mentioned that they are often limited because of time constraints during consultation hours:

On the one hand there are the concerns of the patients, which you have to discuss. But you also have a little bit of pressure, [to tell them] all relevant information. [...] And sometimes it’s already two minutes before the end [of the consultation]. [...] And you can’t just hand out the flyer. You also need to say a few words [about the app] and that’s why I sometimes forgot [to mention the app]. Due to shortage of time.

The nursing experts also mentioned that the nurses oftentimes forgot about the app because it is not part of standard care. Therefore, the nursing experts stated that it might be helpful to better inform the nurses about the app and setting up standards regarding the communication about the app, for example, when to inform the patients and how. In addition, the nursing experts stated that it might be helpful if they had a demonstration device at the oncology unit so that they could better explain the app to the patients. All interviewed health professionals further mentioned that patients with cancer are very diverse and that although some patients are very eager to try out various treatments, others are not. One MBM therapist also stated that not all patients perceive relaxation as important and that those patients might need some additional information which indicates why relaxation is good for them. All health professionals also stated that implementing such an app does not result in a lot of additional work for them and they appreciate the app, which they could recommend to suitable patients.

Regarding their experience with recommending the app to patients, health professionals shared the opinion that female patients are more drawn to mindfulness and relaxation exercises. Furthermore, the MBM therapists stated that patients who already practiced some form of relaxation or meditation often did not participate in the study. The MBM therapists also noticed that the composition of the MBM group had an influence on how many patients were willing to try out the app. For instance, if one patient was very motivated and expressed interest in the app, hesitant patients sometimes followed suit and were willing to try the app as well. One MBM therapist also noticed that many older people were willing to use the app:

I was surprised that so many older patients had the app on their phone and also used the app regularly [...]. I had the impression, that it appeals to the young. [...]. But oftentimes, the older people have more time, because they don’t work anymore.

## Discussion

### Principal Findings

In this study, we explored the feasibility of a mindfulness- and relaxation-based self-help app for patients with cancer. To evaluate the feasibility, we used the RE-AIM framework [37], and in this analysis, we focused on the framework dimensions effectiveness, adoption, and implementation. Our findings support the feasibility of this mHealth intervention. The results indicate that the intervention might have beneficial effects on patients’ distress and quality of life. Furthermore, the mHealth intervention is accepted by the target population as well as by health professionals.

For the dimension effectiveness, we looked into pre-post effects. Our results suggest that the app might have the potential to reduce distress, fatigue, sleep disturbance, and anxiety as well as improve health-related quality of life and mindfulness. This is in line with a recent pilot study [47], in which a mobile mindfulness-based stress reduction program improved, among others, stress, anxiety, depression, sleep quality, quality of life, and mindfulness in patients with breast cancer with small to large effects. Furthermore, a recent randomized controlled trial conducted by Kubo et al [48] assessed the feasibility of a commercially available mindfulness program in which they targeted patients with cancer and their caregivers. This program leads to an increase in quality of life in patients with cancer with a medium effect size [48]. Similar to these findings, Rosen et al [49] reported that the quality of life of patients with breast cancer improved with a small effect size using a commercially available mindfulness course when compared with a control group.

As depressive symptoms and anxiety were not significantly reduced in the total sample in our study, we also looked at subsamples with higher HADS scores. In the high anxiety and high depression subsamples, anxiety and depression, respectively, decreased significantly over time. This might indicate that a mindfulness and relaxation mHealth intervention is especially beneficial for patients with cancer with higher emotional distress. This is also in line with a study by Barth et al [50], where highly distressed patients benefited most from psycho-oncological interventions. However, we did not find any group effects when comparing continuous app users with intervention dropouts. This might indicate that our definition of users and dropouts is not precise enough or that another variable than time spent practicing is responsible for changes in outcomes.

For adoption, our results showed that at week 20 of the intervention, 25 of 100 patients were using the app continuously. With 54 of 100 continuous app users at week 10 [36], this leads to a dropout rate of approximately 50% every 10 weeks. The 25 continuous app users practiced on average 3 to 5 times per week (median), which comes close to our initially stated recommendation of 5 exercises per week. We consider this a good adoption of the mHealth intervention because the intervention was set up as a self-care intervention without the involvement of a therapist or health professional. Mindfulness was the preferred exercise, followed by guided imagery and progressive muscle relaxation. However, mindfulness meditation exercises were also presented as the first choice in the app, whereas guided imagery was placed at the second position, and progressive muscle relaxation was placed at the third position. Therefore, the preference for mindfulness meditation could also be caused by the placement of the exercises in the app. These results regarding adoption are comparable with those of a study conducted by Kubo et al [48], in which patients with cancer received access to the commercially available mindfulness app Headspace (TM). In this study, 40 of 54 patients with cancer allocated to the intervention group completed the 8-week study, and 20 patients with cancer used the app on at least 50% of the days [48].

The results from the interviews with health professionals provide some insights into the implementation of a mindfulness and relaxation mHealth intervention into standard care. In general, all interviewed health professionals perceived the app as a helpful addition to standard care. The health professionals also suggested some improvements, which might increase the acceptance and long-term use of such mHealth interventions by patients. A suggested improvement shared by all health professionals is the increase in the content of the app, such as additional exercises or variations of the exercises. A statement about the implementation of the mHealth intervention given by several health professionals was the adequate provision of information. One of the interviewed MBM psychologists as well as the nursing experts stated that patients with cancer are, on the one hand, flooded with information, especially when they start their treatment. However, the provision of some information to the patients about a mHealth intervention is necessary, at least to let the patients know about the existing intervention. On the other hand, nursing experts also mentioned that nurses often forgot about the intervention, although they approve this kind of intervention. Therefore, a standardized procedure for informing patients about the mHealth intervention might facilitate the implementation of the intervention. In addition, health professionals such as nurses might have to be informed regularly about such interventions because it is not part of their standard treatment; therefore, they might forget about it, as seen in this study. Regarding the recruitment process, the health professionals made the observation that female patients were more interested in this mHealth intervention. This is also reflected by the gender ratio in this study’s sample, with 76 female and 24 male patients with cancer, which is typical for complementary and alternative treatments [51-53]. This gender difference raises the question of whether an effort should be made to better recruit male patients with cancer for such an intervention. A nursing expert, for instance, mentioned during the interview that a focus on more technical aspects or facts could be more appealing to male patients.

### Strengths, Limitations, and Future Directions

This study has several strengths and limitations. A strength of the study is the collection of objective data in the form of logging the exercise use for each patient over the course of 20 weeks. Therefore, data on using the app exercises were not biased through self-report. Another advantage of this study was the use of a mixed methods approach, which is recommended for the development of digital interventions [54].

A limitation of the study is that we did not have a control group. Therefore, the effectiveness of the app cannot be determined in this study because regression to the mean could have an impact on the improvement of well-being. Furthermore, we used paper-and-pencil questionnaires, which might have led to more missing data compared with web-based questionnaires [55]. However, this was compensated by using LMM analyses, which take into account all patients who provided baseline data. Another limitation is that we did not assess whether patients were practicing mindfulness and relaxation exercises without the app, which could have an effect on the assessed outcomes.

Therefore, future studies should investigate this topic with a randomized controlled trial to determine the effectiveness of a mindfulness and relaxation mHealth intervention. Our study provides some insights regarding the effects that might be expected in a similar study, which will be helpful to power future studies sufficiently. We also looked at aspects of implementing an mHealth intervention. All interviewed health professionals perceived such an mHealth intervention as a helpful addition to standard care, but as described earlier, they also stated barriers to the implementation of such an intervention, which should be investigated in future studies. Future studies could also investigate an mHealth intervention with more content than in this study app, as suggested during the interviews by health professionals. For instance, audio files with background music or exercises with variations in their duration could be added. In addition to mindfulness and relaxation exercises, physical exercise programs could be added. Physical exercise can have beneficial effects on symptoms of patients with cancer [56], and physical exercise has already been implemented in mHealth apps for patients with cancer [57].

### Conclusions

The results of this observational feasibility study indicate that a mindfulness and relaxation app can be a feasible and an effective way to deliver a self-care intervention for patients with cancer. Our results indicate that such an intervention might be especially beneficial for highly distressed patients with cancer. The appeal of such an app could be increased with more diverse content, which might also positively affect the adherence of patients to such an intervention. The effectiveness and further aspects regarding the implementation of such an mHealth intervention should be investigated in a future randomized controlled trial.

## Abbreviations

FACT-G: Functional Assessment of Cancer Therapy–General
FMI: Freiburg Mindfulness Inventory
FoP-Q-SF: Fear of Progression Questionnaire-Short Form
HADS: Hospital Anxiety and Depression Scale
LMM: linear mixed model
MBM: mind-body medicine
mHealth: mobile health
PROMIS-29: 29-item Patient-Reported Outcomes Measurement Information System
RE-AIM: Reach, Effectiveness, Adoption, Implementation, and Maintenance
